# A Polymorphism in *Hepatocyte Nuclear Factor 1 Alpha,* rs7310409, Is Associated with Left Main Coronary Artery Disease

**DOI:** 10.1155/2014/924105

**Published:** 2014-08-18

**Authors:** Rui Liu, Hanning Liu, Haiyong Gu, Xiao Teng, Yu Nie, Zhou Zhou, Yan Zhao, Shengshou Hu, Zhe Zheng

**Affiliations:** ^1^State Key Laboratory of Cardiovascular Disease, Fuwai Hospital, National Center for Cardiovascular Diseases, Chinese Academy of Medical Sciences and Peking Union Medical College, 167 Beilishilu Street, Beijing 100037, China; ^2^Department of Cardiothoracic Surgery, Affiliated People's Hospital of Jiangsu University, Zhenjiang 212000, China

## Abstract

Coronary artery disease is the leading cause of mortality and morbidity in the world. Left main coronary artery disease (LMCAD) is a particularly severe phenotypic form of CAD and has a genetic basis. We hypothesized that some inflammation- and hyperhomocysteinemia-related gene polymorphisms may contribute to LMCAD susceptibility in a Chinese population. We studied the association between polymorphisms in the genes hepatocyte nuclear factor 1 alpha (HNF1A; rs7310409, G/A), C-reactive protein (rs1800947 and rs3093059 T/C), methylenetetrahydrofolate reductase (rs1801133, C/T), and methylenetetrahydrofolate dehydrogenase (rs1076991, A/G) in 402 LMCAD and 804 more peripheral CAD patients in a Chinese population. Genotyping was performed using the matrix-assisted laser desorption/ionization time-of-flight mass spectrometry method. When the HNF1A rs7310409 GG homozygote genotype was used as the reference group, both the individual, GA and AA, and combined GA/AA genotypes were associated with an increased risk of LMCAD. This single nucleotide polymorphism (rs7310409) is strongly associated with plasma CRP levels. In conclusion, the present study provides evidence that the HNF1A rs7310409 G/A functional polymorphism may contribute to the risk of LMCAD.

## 1. Introduction

Coronary artery disease (CAD) is the leading cause of mortality and morbidity in the world [1, page e18–e209]. CAD manifests multifaceted phenotypes, including variations in the number of vessels involved, the location of lesions, and the severity of vessel diameter narrowing. These differences are considered to result from the complex interaction of numerous environmental and genetic factors.

Left main coronary artery disease (LMCAD) is a particularly severe phenotypic variant of CAD. The left main coronary artery (LMCA) arises from the left aortic sinus and extends to the bifurcation into the left anterior descending and left circumflex arteries. This vessel is responsible for more than 75% of the blood supply to the left ventricular cardiac mass in patients with right dominant or balanced type cardiac circulation and 100% in those with left dominant type. Therefore, in the case of a lesion to the LMCA, the left ventricle (LV) will receive less blood supply and present impaired function, leading to a sharply increased risk of life-threatening events caused by LV dysfunction and arrhythmias in patients [2, page 36–50b]

Previous studies suggest that CAD lesions with different morphologies display distinct degrees of heritability, and lesions in the locations considered most hazardous, such as LMCAD, are much more frequently shared by siblings with CAD [3, 4] (pages 855–862; 2432–2437). Further studies have provided evidence that LMCAD shows a high degree of heritability, and several single nucleotide polymorphisms (SNPs) associated with LMCAD have been identified [5–7] (pages 443–453; 156; 461–465).

Inflammation plays an important role in the initiation, progression, and clinical outcomes of CAD and other manifestations of atherosclerosis [8, pages 1685–1695]. C-reactive protein (CRP) is a pattern-recognition molecule of the innate immune system, an acute phase reactant, and a hallmark of low-grade systemic inflammation [9, pages 237–242]. CRP is also one of the many molecular factors involved in the pathogenesis of CAD, with elevated CRP plasma levels associated with an increased risk of cardiovascular events [10, pages 96–102]. Recent investigations have revealed significant associations of SNPs in* hepatocyte nuclear factor 1 alpha* (*HNF1A*) with CRP levels [11, pages 244–254]. The* HNF1A* gene localizes to chromosome 12q, comprises ten exons, spans ~23 kb, and encodes a transcription factor that is expressed in many different tissues [12, 13] (pages 4199–4204; 575–585). Two genome-wide association studies have provided evidence that SNPs within a 5 kb region of* HNF1A* intron 1 are strongly associated with plasma CRP levels [14, 15] (pages 1193–1201; 1185–1192), and one of these SNPs, rs7310409, presents the strongest association [15, pages 1185–1192].

A high homocysteine level is a further independent risk factor for cardiovascular disease [16, 17] (pages 2015–2022; 1202). Methylenetetrahydrofolate dehydrogenase (MTHFD) and 5,10-methylenetetrahydrofolate reductase (MTHFR) are two important folate-metabolizing enzymes involved in the folate/homocysteine metabolic pathway. The* MTHFR* gene is located on chromosome 1p36.3 and encodes an enzyme that is essential for folate-mediated one-carbon metabolism.* MTHFD* localizes to chromosome 14 (14q24) and encodes a protein with catalytic activities playing indirect roles in folate and homocysteine metabolism by contributing to the 5,10-MTHFR pool.

On the basis of the biological and pathological significance of HNF1A, CRP, MTHFR, and MTHFD, it is possible that functional genetic variations in these genes may contribute to the phenotype of CAD. The objective of this investigation was to evaluate the associations between* HNF1A* rs7310409 G/A,* CRP* rs1800947 G/C,* CRP* rs3093059 T/C,* MTHFR* rs1801133 C/T, and* MTHFD* rs1076991 A/G polymorphisms and LMCAD susceptibility in a hospital-based study with a case-case design. We genotyped these five SNPs in a cohort of 1206 Chinese patients, in which 402 patients with LMCAD and 804 with more peripheral coronary artery disease (MPCAD) were included.

## 2. Methods

### 2.1. Study Subjects

The study protocol was approved by the Review Board of Peking Union Medical College (Beijing, China). This study complied with the World Medical Association Declaration of Helsinki regarding the ethical conduct of research involving human subjects and/or animals. Every subject provided written informed consent to be recruited in the study.

This study involved 1206 patients with CAD. Patients were consecutively recruited from the Cardiovascular Institute and Fuwai Hospital, Chinese Academy of Medical Sciences, and Peking Union Medical College (Beijing, China), between December 2007 and December 2008. All patients were diagnosed using angiography. LMCAD was defined as a lesion compromising the lumen by >50%, proximal to the bifurcation, including ostial stenosis. Lesions compromising the lumen by >50% outside of the LMCA were defined as MPCAD.

All subjects were genetically unrelated ethnic Han Chinese. All data were collected by trained clinical research staff and were subsequently double-entered into computer databases. Baseline information on personal and clinical characteristics was complete for all 1206 patients involved in the study.

### 2.2. Isolation of DNA and Genotyping with Matrix-Assisted Laser Desorption/Ionization Time-of-Flight (MALDI-TOF) Mass Spectrometry

**Figure 1:**
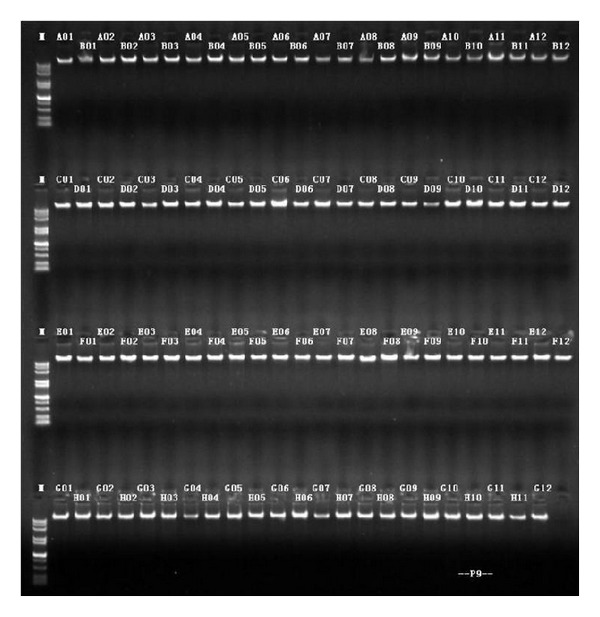
PCR-amplified DNA analyzed on a 3% agarose gel and visualized by ethidium bromide staining.

**Figure 2:**
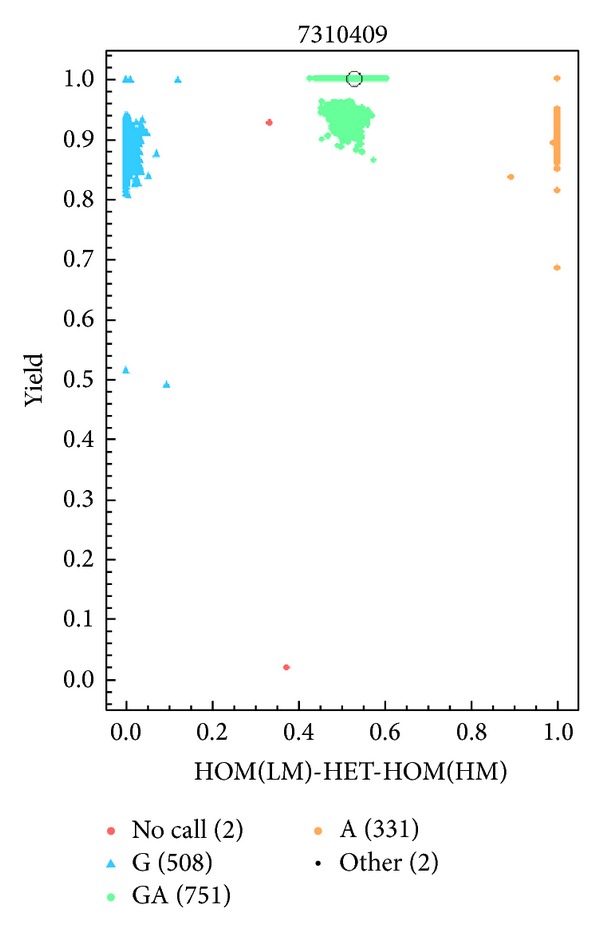
Genotyping of* HNF1A* rs7310409 G/A by MALDI-TOF mass spectrometry.

Blood samples were collected into vacutainers and then transferred to test tubes which contain ethylenediaminetetraacetic acid. The Wizard Genomic DNA Purification Kit (Promega, Madison, WI) was used to isolate genomic DNA from whole blood. For quality control, a polymerase chain reaction (PCR) was conducted using all DNA samples, which was analyzed on a 3% agarose gel and visualized by ethidium bromide staining (Figure 1).* HNF1A* rs7310409 G/A,* CRP *rs1800947 G/C,* CRP *rs3093059 T/C,* MTHFR* rs1801133 C/T, and* MTHFD* rs1076991 A/G genotyping were performed by MALDI-TOF mass spectrometry, and the MassARRAY system (Sequenom, San Diego, CA, USA) was used as previously described [18, pages 1637–1647] with support from Capital Bio Corporation (Beijing, China). LMCAD (*n* = 402) and MPCAD (*n* = 804) samples were assayed at a ratio of 1 : 2. Completed genotyping reactions were spotted into a 384-well spectroCHIP (Sequenom) with a MassARRAY Nanodispenser (Sequenom) and analyzed by MALDI-TOF mass spectrometry. MassARRAY RT software (version 3.1; Sequenom) was used to perform genotype calling in real time and the analysis was made by MassARRAY Typer software (version 4.0; Sequenom; Figure 2).

### 2.3. Statistical Analyses

A chi-squared test was used to evaluate the differences in demographic variables, clinical features, and genotypes of the five SNP variants. The associations between the five SNPs and risk of CAD phenotypes were estimated by computing the odds ratios (ORs) and their 95% confidence intervals (CIs) using logistic regression analyses for crude ORs and adjusted ORs when adjusting for age and sex. Statistical analyses were performed by SAS software (version 9.1.3; SAS Institute, Cary, NC, USA).

## 3. Results

### 3.1. Characteristics of the Study Population

Among 1206 DNA samples, the genotyping success rate for the five SNPs ranged from 98.83% to 99.92%. The demographic and clinical characteristics of all the subjects are presented in Table 1. The mean age of LMCAD patients (62.24 ± 8.66 years) was significantly higher than that of MPCAD patients (60.14 ± 8.96 years), *P* < 0.001 (Table 1). There were no significant differences between the two patient groups for sex, mean body mass index, family history of CAD, previous smoking, hypertension, hyperlipidemia, diabetes mellitus, mean ejection fraction, circumflex branch of left coronary artery system, right coronary artery system, or OPCAB (off-pump coronary artery bypass grafting)/cCABG (conventional coronary artery bypass grafting) (Table 1). LMCAD patients were significantly more likely to have three disease territories than those with MPCAD (93.3% versus 87.7%, *P* = 0.003) (Table 1).

### 3.2. Associations between the Five SNPs and the Risk of LMCAD and MPCAD

The information about the five SNPs was listed in Table 2. The genotype frequencies of the* HNF1A* rs7310409 G/A polymorphism were 27.4% (GG), 50.1% (GA), and 22.4% (AA) in LMCAD patients and 35.1% (GG), 45.0% (GA), and 19.9% (AA) in MPCAD patients (*P* = 0.027) (Table 3). When the* HNF1A* rs7310409 GG homozygote genotype was used as the reference group, the GA genotype was associated with an increased risk of LMCAD (GA versus GG, adjusted OR = 1.46, 95% CI = 1.10–1.93, *P* = 0.009). The AA genotype was also associated with an increased risk of LMCAD (AA versus GG, adjusted OR = 1.45, 95% CI = 1.03–2.04, *P* = 0.033). In the dominant model, when the* HNF1A* rs7310409 GG genotype was used as the reference group, the combined GA/AA genotypes were associated with an increased risk of LMCAD (GA/AA versus GG, adjusted OR = 1.45, 95% CI = 1.17–1.89, *P* = 0.006). The polymorphism was not associated with the risk of LMCAD in a recessive genetic model (AA versus GG/GA, adjusted OR = 1.16, 95% CI = 0.86–1.55, *P* = 0.331).

None of the other four SNPs demonstrated a significant difference in genotype distributions between LMCAD and MPCAD in any comparison model (Table 3). After the Bonferroni correction, for* HNF1A *rs7310409 G/A, *P*_correct_ = 0.045 for GA versus GG, *P*_correct_ = 0.165 for AA versus GG, *P*_correct_ = 1.000 for AA versus GG/GA, and *P*_correct_ = 0.030 for GA/AA versus GG after being adjusted for age and sex. For the rest of the 4 SNPs, *P*_correct_ > 0.05 in all comparison models.

## 4. Discussion

We investigated the associations between the SNPs* HNF1A* rs7310409 G/A,* MTHFR* rs1801133 C/T,* CRP* rs1800947 G/C,* CRP* rs3093059 T/C, and* MTHFD* rs1076991 A/G and the risk of LMCAD in a Chinese population and found that the* HNF1A* rs7310409 G/A polymorphism may increase the risk of LMCAD.

Many previous studies have suggested that LMCAD has a high heritable component. Nine of twelve monozygotic twin pairs displayed concordance for LMCAD, whereas only three of twelve were concordant for MPCAD [19, 20] (pages 91–96; 193–197). In addition, the first centimeters of both the LMCA and the right coronary artery are naturally resistant to atherosclerosis. This resistance might be regarded as an inherited characteristic, influencing the process of coronary atherosclerosis, particularly of clinically significant lesions [21, pages 173–181]. Fischer et al. reported that LMCAD displays higher heritability than MPCAD, but the reason for this difference remains to be elucidated [3, pages 855–862]. One possible explanation is that the ontogenesis of the proximal parts of coronary arteries and other coronary arteries is different. The proximal portion of coronary arteries buds from the walls of the truncus arteriosus, while the more peripheral portion develops as a subepicardial vascular network [3, pages 855–862]. Some studies concerning the relationship between certain SNPs and LMCAD have provided more direct evidence that LMCAD is heritable. Wang et al. reported the first genetic association study focusing on LMCAD. They analyzed 102 LMCAD cases and 149 controls and found that polymorphisms in an intron of* limbic system-associated membrane protein*, a tumor suppressor gene, are associated with LMCAD [5, pages 443–453]. Bousoula et al. studied SNPs in genes related to prostaglandin I_2_ and prostacyclin synthesis in 151 LMCAD cases and 103 controls and elucidated that the CC genotype of the C1117A polymorphism is associated with a higher risk of LMCAD [7, pages 461–465]. Kolovou et al. investigated* cholesteryl ester transfer protein* (*CETP*) polymorphisms in 133 subjects with angiographically documented LMCAD, 241 subjects with MPCAD, and 97 controls. However, they found no significant difference in CETP allele frequencies or genotype distributions among LMCAD and MPCAD patients. Notably, in contrast to our study, all of these studies used people without CAD as controls. All of our research subjects were CAD patients, indicating that our results demonstrate the relationship between the genotype distributions among CAD patients with LMCAD and MPCAD phenotypes [6, page 156].

Inflammation is involved in all stages of the development of atherosclerosis and is both a cause and a consequence of ischemic heart disease. CRP is a pattern-recognition molecule of the innate immune system, an acute phase reactant, and a hallmark of low-grade systemic inflammation. Prospective studies indicate an association between the level of CRP and the long-term risk of cardiovascular disease [9, pages 237–242]. High plasma levels of CRP independently predict incident metabolic syndrome [22, pages 2016–2021], type 2 diabetes [23, pages 327–334], myocardial infarction [24, pages 1557–1565], and stroke, and variation in CRP is heritable [25, pages 1115–1122].

*HNF1A*, which encodes a transcription factor, is expressed in many different tissues including the liver, alimentary tract, kidney, and pancreas [12, 13] (pages 4199–4204; 575–585). The* HNF1A* rs7310409 SNP is located within a 5 kb region spanning the 3′ half of the first intron of* HNF1A*, and this was the typed* HNF1A* SNP that was most strongly associated with CRP in the Women's Genome Health Study [15, pages 1185–1192]. The human CRP promoter contains two functional HNF1A-binding sites, and HNF1A is necessary for the induction of CRP [26, pages 4467–4475].* HNF1A* polymorphisms have a significant impact on CRP levels [15, pages 1185–1192] and are independently associated with CRP levels in the Taiwanese population [27, pages 725–729]. In a meta-analysis on the effect of 12 SNPs within 8 candidate loci in 36,752 Asians,* HNF1A *rs7310409 G/A (*P* = 3.4∗10^−23^) had the most significant effects on CRP levels [28]. Thus,* HNF1A*SNPs may influence CRP levels and play a role in LMCAD etiology.

Considering the frequency of* HNF1A* rs7310409 G/A alleles in the LMCAD group, assuming an adjusted OR of 1.45 and the number of LMCAD and MPCAD samples is *n* = 401 and 803, respectively, the power of our analysis (*α* = 0.05) was 0.858.

To our knowledge, the present study is the first to focus on SNPs involved in two important independent risk factors for CAD phenotypes (LMCAD and MPCAD), inflammation and hyperhomocysteinemia. Moreover, this investigation recruited the largest cohort of patients with LMCAD ever reported among studies of SNP associations with this phenotype.

Several limitations of the present study need to be addressed. First, this was a hospital-based study; therefore, selection bias was unavoidable. Second, the polymorphisms we investigated, based on functional considerations, may not offer a comprehensive view of the genetic variability underlying these phenotypes. Fine-mapping and even high-density whole genomegenetic analyses evaluating different CAD phenotypes might give further insights into the pathophysiological mechanisms underlying LMCAD. Third, a single case-case study is insufficient to fully interpret the relationship between* HNF1A* rs7310409 G/A and susceptibility to LMCAD, given the moderate number of patients included. Replication studies with larger numbers of subjects are necessary to confirm our findings. Finally, we did not evaluate plasma CRP levels or the function of* HNF1A* rs7310409 G/A, which restricted our analyses.

In conclusion, this study provides strong evidence that the* HNF1A* rs7310409 G/A functional polymorphism may contribute to the risk of developing the LMCAD phenotype. However, our results were obtained from a moderately sized sample; therefore, this is a preliminary conclusion. Validation by a larger study and in more ethnically diverse populations is needed to confirm these findings.

## Figures and Tables

**Table 1:** Patient demographics and risk factors for CAD (LMCAD and MPCAD) (all subjects).

Variable	LMCAD^b^ (*n* = 402)	MPCAD^c^ (*n* = 804)	*P* value	All CAD^d^ (*n* = 1206)
Mean age, years	62.24 (±8.66)	60.14 (±8.96)	**<0.001**	60.84 (±8.91)
Female, %	67 (16.7)	170 (21.1)	0.065	237 (19.7)
Mean BMI^a^, kg/m^2^	25.55 (±3.16)	25.75 (±3.13)	0.299	25.68 (±3.14)
Family history of CAD, %	21 (5.2)	32 (4.0)	0.323	53 (4.4)
Previous smoker, %	211 (52.5)	422 (52.5)	1.000	633 (52.5)
Hypertension, %	261 (64.9)	530 (65.9)	0.732	791 (65.6)
Hyperlipidemia, %	275 (68.4)	573 (71.3)	0.290	848 (70.4)
Diabetes mellitus, %	125 (31.1)	271 (33.7)	0.363	396 (32.8)
Mean ejection fraction, %	60.25 (±7.96)	59.55 (±8.76)	0.177	59.78 (±8.50)
Disease territories, %				
1-2	27 (6.7)	99 (12.3)	**0.003**	126 (10.4)
3	375 (93.3)	705 (87.7)	**0.003**	1080 (89.6)
LMCAD, %	402 (100)	0 (0)	—	402 (33.3)
Anterior descending artery system, %	386 (96.0)	793 (98.6)	**0.004**	1179 (97.8)
Circumflex branch of left coronary artery system, %	373 (92.8)	744 (92.5)	0.876	1117 (92.6)
Right coronary artery system, %	374 (93.0)	751 (93.4)	0.807	1125 (93.3)
OPCAB^e^/cCABG^f^	209/193	438/366	0.414	647/559

^a^BMI: body mass index; ^b^LMCAD: left main coronary artery disease; ^c^MPCAD: more peripheral coronary artery disease. Bold values are statistically significant (*P* < 0.05); ^d^CAD: coronary artery disease; ^e^OPCAB: off-pump coronary artery bypass grafting; ^f^cCABG: conventional coronary artery bypass grafting.

**Table 2:** Primary information for five genotyped SNPs.

Genotyped SNP	Chr^a^	Regulome DBscore^b^	TFBS^c^	Splicing (ESE or ESS)	Location	MAF^d^ for Chinese population in database	Genotyping value, %
*HNF1A*, rs7310409 G/A	12	5	—	—	Intron1	0.366	99.83
*CRP*, rs1800947 G/C	1	6	—	—	Synonymous	0.067	99.92
*CRP*, rs3093059 T/C	1	No data	Y	—	5′ Near gene	0.171	99.83
*MTHFR*, rs1801133 C/T	1	4	—	—	Missense	0.341	99.83
*MTHFD*, rs1076991 A/G	14	4	Y	—	5′ UTR	0.317	99.83

^a^Chr: chromosome; ^b^DBscore: http://www.regulomedb.org/; ^c^TFBS: transcription factor binding site (http://snpinfo.niehs.nih.gov/snpinfo/snpfunc.htm); ^d^MAF: minor allele frequency.

**Table 3:** Main effects of SNPs on LMCAD risk.

Genotyped SNP	Genotyping (AA/AB/BB)^a^		AB versus AA adjusted OR (95% CI); *P* value	BB versus AA adjusted OR (95% CI); *P* value	BB versus (AA + AB) adjusted OR (95% CI); *P* value	(BB + AB) versus AA adjusted OR (95% CI); *P* value	*P*-trend
	LMCAD (*n* = 402)	MPCAD (*n* = 804)					
*HNF1A*: rs7310409 G/A	110/201/90	282/361/160	**1.46 (1.10–1.93); 0.009**	**1.45 (1.03–2.04); 0.033**	1.16 (0.86–1.55); 0.331	**1.45 (1.17–1.89); 0.006**	**0.027**
*CRP*: rs1800947 G/C	367/34/0	745/58/1	1.20 (0.77–1.87); 0.417	—; 0.981	—; 0.981	1.18 (0.76–1.84); 0.458	0.578
*CRP*: rs3093059 T/C	282/102/17	552/231/20	0.88 (0.67–1.16); 0.365	1.68 (0.86–3.28); 0.127	1.74 (0.90–3.38); 0.100	0.95 (0.73–1.23); 0.671	0.147
*MTHFR*: rs1801133 T/C	133/199/69	291/372/140	1.15 (0.88–1.51); 0.298	1.07 (0.75–1.52); 0.725	0.98 (0.71–1.35); 0.905	1.13 (0.88–1.46); 0.347	0.512
*MTHFD*: rs1076991 A/G	217/151/33	434/302/67	0.98 (0.76–1.27); 0.876	0.97 (0.62–1.52); 0.887	0.98 (0.63–1.51); 0.914	0.98 (0.77–1.25); 0.855	0.998

^a^AA/AB/BB are dominant homozygote, heterozygote, and recessive homozygote, respectively; bold values are statistically significant (*P* < 0.05); Bonferroni correction was performed to correct the *P* value (*P*_correct_); for *HNF1A* rs7310409 G/A, *P*_correct_ = 0.045 for GA versus GG, *P*_correct_ = 0.165 for AA versus GG, *P*_correct_ = 1.000 for AA versus GG/GA, and *P*_correct_ = 0.030 for GA/AA versus GG after being adjusted for age and sex. For the rest of the 4 SNPs, *P*_correct_ > 0.05 in all comparison models, adjusted for age and sex.
